# How good are clinicians in predicting the presence of *Pseudomonas* spp. in diabetic foot infections? A prospective clinical evaluation

**DOI:** 10.1002/edm2.225

**Published:** 2021-02-09

**Authors:** Ilker Uçkay, Dominique Holy, Madlaina Schöni, Felix W. A. Waibel, Tudor Trache, Jan Burkhard, Thomas Böni, Benjamin A. Lipsky, Martin C. Berli

**Affiliations:** ^1^ Infectiology Balgrist University Hospital Zurich Switzerland; ^2^ Department of Orthopedic Surgery Balgrist University Hospital Zurich Switzerland; ^3^ Internal Medicine Balgrist University Hospital Zurich Switzerland; ^4^ Department of Medicine University of Washington Seattle WA USA

**Keywords:** clinical prediction, diabetic foot infections, *Pseudomonas aeruginosa*

## Abstract

**Introduction:**

The most frequently prescribed empirical antibiotic agents for mild and moderate diabetic foot infections (DFIs) are amino‐penicillins and second‐generation cephalosporins that do not cover *Pseudomonas* spp. Many clinicians believe they can predict the involvement of *Pseudomonas* in a DFI by visual and/or olfactory clues, but no data support this assertion.

**Methods:**

In this prospective observational study, we separately asked 13 experienced (median 11 years) healthcare workers whether they thought the *Pseudomonas* spp. would be implicated in the DFI. Their predictions were compared with the results of cultures of deep/intraoperative specimens and/or the clinical remission of DFI achieved with antibiotic agents that did not cover *Pseudomonas*.

**Results:**

Among 221 DFI episodes in 88 individual patients, intraoperative tissue cultures grew *Pseudomonas* in 22 cases (10%, including six bone samples). The presence of *Pseudomonas* was correctly predicted with a sensitivity of 0.32, specificity of 0.84, positive predictive value of 0.18 and negative predictive value 0.92. Despite two feedbacks of the interim results and a 2‐year period, the clinicians' predictive performance did not improve.

**Conclusion:**

The combined visual and olfactory performance of experienced clinicians in predicting the presence of *Pseudomonas* in a DFI was moderate, with better specificity than sensitivity, and did not improve over time. Further investigations are needed to determine whether clinicians should use a negative prediction of the presence of *Pseudomonas* in a DFI, especially in settings with a high prevalence of pseudomonal DFIs.

## Graphical abstract

The clinical diagnosis of *Pseudomonas* infection in the diabetic foot is difficult. Even very experienced colleagues (surgeons, infectious diseases physicians) need the support of a good microbiological sample.

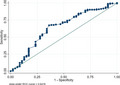

## 1 INTRODUCTION

Diabetic foot infections (DFIs), including diabetic foot osteomyelitis (DFO), are common and associated with substantial morbidity, costs and antibiotic use.^1^, ^2^, ^3^ When clinicians face the choice of selecting an initial empirical antibiotic regimen for most mild and moderate DFIs,^4^, ^5^ one pathogen has exceptional prominence in their judgement: *Pseudomonas aeruginosa*.^6^, ^7^ This is because they perceive *Pseudomonas* to be both a common and a highly antibiotic‐resistant pathogen. In fact, microbiological surveys from around the world have shown that it is a frequent isolate from DFIs in subtropical regions (eg South [Eastern] Asia or the Middle East), but far less so in temperate areas (eg North America and Europe).^2^, ^5^ These studies have confirmed that *P*.* aeruginosa* is naturally resistant to standard antibiotics^4^ most often prescribed for mild and moderate DFIs,^4^, ^5^ such as amino‐penicillins or first‐ and second‐generation cephalosporins. The guidelines on DFI published by both the Infectious Diseases Society of America (IDSA)^4^ and the International Working Group on the Diabetic Foot (IWGDF)^5^ recommend selecting empiric anti‐pseudomonal antibiotic agents only when *P*.* aeruginosa* is a documented pathogen, in settings where it has a high prevalence, or an empirical coverage in virulent, acute severe infections such as in sepsis.^5^

Many such recommendations, however, presume that clinicians are able to judge the likelihood of the presence of *Pseudomonas* in an individual patient. While knowing certain clinic‐demographic information (eg geographical location, previous antimicrobial treatments or surgery) is likely useful in judging the pre‐culture likelihood of *Pseudomonas*, little is known about the accuracy of clinical diagnosis. Nonetheless, many surgeons, internists, podiatrists and specialized nurses believe that they are able to predict *Pseudomonas* spp. by detecting certain visual (green colour^8^) and/or olfactory (grape fruit–like smell^9^) clues. There are, however, no data to support this widespread assumption. If accurate, using these quick, convenient and inexpensive clinical findings could be a major help in avoiding antibiotic therapy that is either unnecessarily broad‐spectrum or that fails to cover the causative pathogen, at least for mild and moderate DFIs. Thus, we undertook a prospective observational study to assess the clinical performance of various healthcare providers in our specialized, tertiary centre for DFIs in predicting the clinical involvement of *Pseudomonas* on infected wound culture. Of note, we do not analyse the impact of *P*.* aeruginosa* on the DFI outcomes, or the prediction of pseudomonal colonization in diabetic foot ulcers, for which a broader literature is available.^6^, ^7^

## 2 METHODS

### 2.1 Setting

The Balgrist University Hospital in Zurich is affiliated with the University of Zurich and is a tertiary referral centre for patients with DFIs. For these patients, it employs a multidisciplinary team composed of four diabetic foot surgeons, three internists, a hospital pharmacist, five specialized wound nurses, radiologist experts in musculoskeletal disorders, a diabetes nurse, three nutritionists, an orthopaedic shoe factory, prosthesis specialists and an infectious diseases physician specialized in orthopaedic infections.^10^

### 2.2 Study population, study conduct and criteria

**FIGURE 1:**
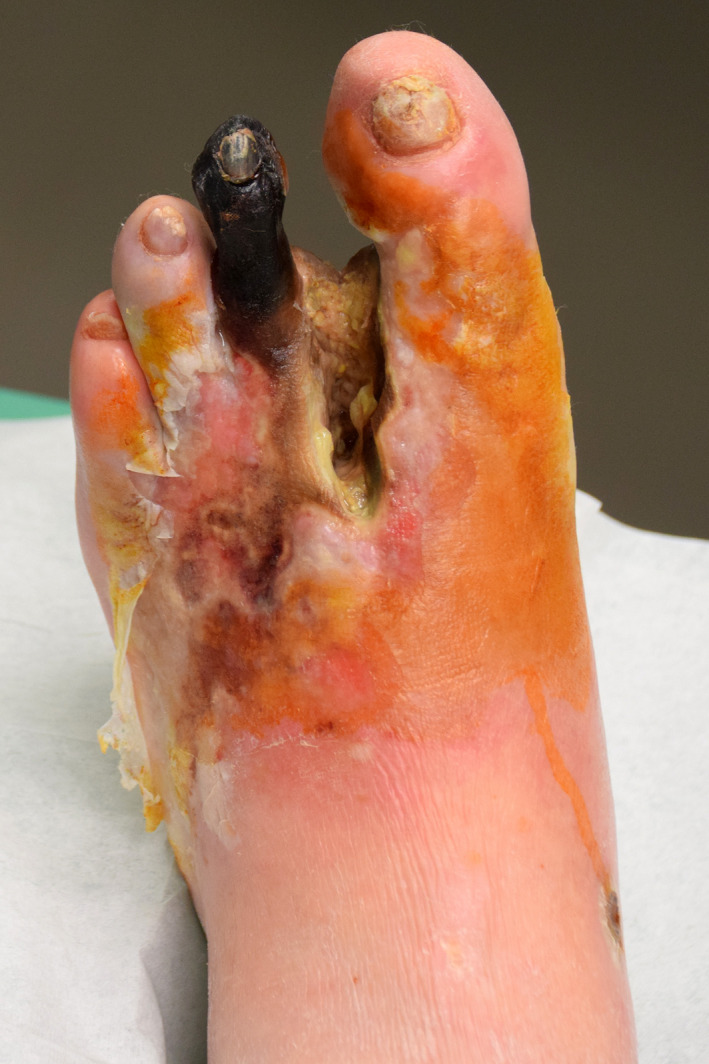
Photograph of a diabetic patient with mixed infection of the foot due to *Pseudomonas aeruginosa* and three other pathogens. Please note the absence of a clear green colour around the infected and ischaemic skin. The colour is rather yellowish. *Permitted by patient*

Enrolment in this study began on 10 August 2018 and was terminated on 20 August 2020. During this 2‐year period, we asked the experienced healthcare workers (HCWs) of our DFI team to predict whether or not they thought that *Pseudomonas* was involved as a causative organism in every DFI episode admitted to our centre. Only HCWs with at least 1 year of 100% daily experience on the DFI team were allowed to participate. HCWs could use their own subjective definition of visual clues (eg greenish colour^8^) (Figure 1) or olfactory clues (eg grape juice–like smell^9^). Importantly, to ensure that HCW only expressed their own opinion, we solicited their responses individually. HCW provided their prediction before results of the Gram staining or of the intraoperative, deep tissue microbiological cultures were available, and we excluded cases with known microbiological results. The HCW was, however, allowed to know the actual empirical antibiotic regimen selected by the referring general practitioner and could also use the presence or absence of maceration or local ischaemia as a guide to predict the presence of *Pseudomonas*. In our clinical experience and according to a widespread thinking in the world, *Pseudomonas* spp. would not cause maceration by itself, but chronic maceration can become the habitat of nonfermenting rods^11^ as *Pseudomonas* spp. or colonized by (Gram‐negative) anaerobes.^12^ Our criterion standard to judge the prediction was the presence or absence on microbiological clinical cultures of *Pseudomonas spp*, and/or the patient achieving clinical remission of DFI when treated only with antibiotic agents that were not active against *Pseudomonas*. Assessing the clinical evolution of the infection was an integral part of the study. For example, if the cultures grew *P*.* aeruginosa*, among multiple other organisms, and the patient was cured with co‐amoxiclav alone (without radical amputation),^13^, ^14^ we did not consider the *Pseudomonas* a pathogen. We defined the presence of DFI by the IDSA criteria^4^ and performed microbiological assessments by standard techniques, based on the EUCAST recommendations.^15^ Our Microbiology Laboratory routinely seeks and reports about *Pseudomonas* spp. in cases of polymicrobial DFI.

We recorded not only the HCW's “yes or no” prediction of the presence of *Pseudomonas* on an Excel™ file, but also any empirical antibiotic therapy administered prior to collecting microbiological samples, as well as the sex of the patient and participating HCW. Appendix S1 resumes the variables that we noted directly on the Excel™ file. This is because several studies have found that olfactory abilities of women are superior to those of men.^16^, ^17^ Moreover, we evaluated whether there was an effect of the result of finding of Gram‐negative organisms on a stained smear (before the availability of the microbiological cultures) on pseudomonal DFI, independently of the prior prediction by the HCWs. Because the participating HCW had no chance to know the Gram‐stained smear at the time of prediction, this additional analysis was hypothetical in terms of the real‐life practice during our study.

After an initial assessment of the baseline prediction performance of the involved HCW during the first 6 months of the study, we presented the interim results after 85 predictions on 10 February 2020 to them. This was mostly given orally but was accompanied by distributing to them an abstract detailing the findings for the Swiss National Congress 2020. We gave the surgeons a second presentation of the results in form of a lecture using a PowerPoint™ presentation (that we mandated they attend) after we enrolled 160 episodes on 20 July 2020 (Appendix S2). Furthermore, we gave the HCW continuous verbal feedback on the preliminary results at the bed‐side during the medical visits. This evaluation was conducted as part of the larger “DF‐MANAG” study conglomerate that evaluates clinical, laboratory and radiological variables associated with various outcomes in the management of the diabetic foot syndrome (Ethical Committee Zurich, BASEC number 2019‐01994). Many of the patients whose results were used in this study also participated in one or both of two randomized controlled DFI trials on the duration of antibiotic therapy (ClinicalTrial.gov NCT04081792; BASEC number 2019‐00778).^10^

### 2.3 Statistical analyses

The primary outcome of interest was the sensitivity, specificity, and positive and negative predictive values^18^ of the accuracy of the clinical prediction by HCW of the presence *P*.* aeruginosa* in DFIs. As secondary outcomes, we stratified these results for seven individual substrata: (1) prediction before and after the interim results presentations; (2) prediction by just the surgeons alone; (3) prediction of female (vs male) HCWs; (4) prediction based on the presence of Gram‐negative rods on the stained specimens; (5) prediction based on the presence of DFO; (6) visual predictions only; and (7) olfactory predictions only. In contrast to case‐control studies or randomized trials, the sample size requirements for diagnostic tests are flawed, difficult to compute and based on experiences with previous studies and the overall prevalence of the key variable, that is *P*.* aeruginosa*. With an estimated *P*. *aeruginosa* prevalence of 12% in our centre (7%–15% for Switzerland^2^, ^3^, ^11^, ^14^, ^19^), we targeted a minimal number of 200 predictions for our evaluation. We predicted that this size would provide one dozen episodes of true pseudomonal DFIs and 180–190 nonpseudomonal DFI controls, which we considered sufficient to compute the predictive value of a diagnostic guess.^18^

We compared groups using the Pearson chi‐square test and evaluated changes in the prediction performance over time with the P‐for‐trend test. We performed an unconditional, multivariate logistic regression analysis to determine associations with the outcome “correct prediction,” which included the true‐positive and the true‐negative results. In this multivariate analysis, we also included an additional (continuous) variable: the number of years each HCW had daily professional experience in the field of DFI management. We introduced all independent variables into the multivariate analysis and checked for collinearity and effect modification with interaction terms. We used STATA™ software (15.0) and considered *p* values ≤ .05 (two‐tailed) as significant. The STATA™ command “lroc” printed the receiver operating characteristic (ROC) curve regarding the accuracy of our predictions.

## 3 RESULTS

### 3.1 Healthcare workers

Overall, 13 different specialized HCWs (six nurses, four orthopaedic surgeons, two internists and one infectious diseases specialist), seven of whom were female, participated in the study. Their median‐weighted number of years with full professional activity in the field of DFI was 11 years (range, 1–30 years). Overall, 65 predictions were made by female HCWs (22% of the total, with more nurses than physicians) and 88 by orthopaedic surgeons (40%; one female). The visual and olfactory predictions paralleled each other in 38 evaluations (Spearman rho correlation coefficient 69%, *p* < .01), with more visual (*n* = 38; 38/221; 17%) than olfactory predictions (*n* = 28; 13%). The proportions of olfactory predictions in terms of suspected *P*.* aeruginosa* were similar between female and male HCWs (10/55 vs 18/138; *p* = .43). Based on our individual interviews of participants, the most frequent elements they used in favour of the presence of *Pseudomonas* spp. were a green colour in the wound or macerated skin. The presence of a characteristic smell was the least cited element. This order did not change after our presentation to the HCWs of the preliminary results.

### 3.2 Patients and infections

We included 221 DFI evaluations (121 of which were episodes of DFO) that occurred in 88 individual adult patients (41 (19%) of which were in female patients), with and without concomitant foot ischaemia. Culture specimens grew *P*. *aeruginosa* in 22 cases (overall prevalence 10%; six were DFOs and six were monomicrobial infections). All monomicrobial infections due to *Pseudomonas* were from a specimen of bone (ie cases of DFO). In the 100 exclusively soft‐tissue infections, only four *Pseudomonas* cases demonstrated a green colour on the wound or the dressing, and most of these were more yellow than green. There were a total of 62 different microbiological results, with the most frequent groups being *Staphylococcus aureus* (*n* = 82; 37%), streptococci (*n* = 28; 13%), enterococci (*n* = 27; 12%) and *Enterobacter* spp, (*n* = 18; 8%). Overall, specimens from 51 DFIs (23%) grew Gram‐negative bacteria. The delay in return of the microbiological results lagged between 2 and 4 days. This delay was indifferent for cultures with and without pseudomonal involvement. In 77 episodes (77/221; 35%), the patients were taking empirical oral antibiotic therapy before the wound sampling, including the following: co‐amoxiclav (*n* = 51; 66%),^3^ levofloxacin (*n* = 16; 21%), clindamycin (*n* = 4; 5%) or other antibiotic combinations. The isolated pathogens were susceptible to this pre‐sampling antibiotic agent in 58 episodes (58/77; 75%) and resistant in 19 cases (25%).

### 3.3 Performances of the predictions

**FIGURE 2:**
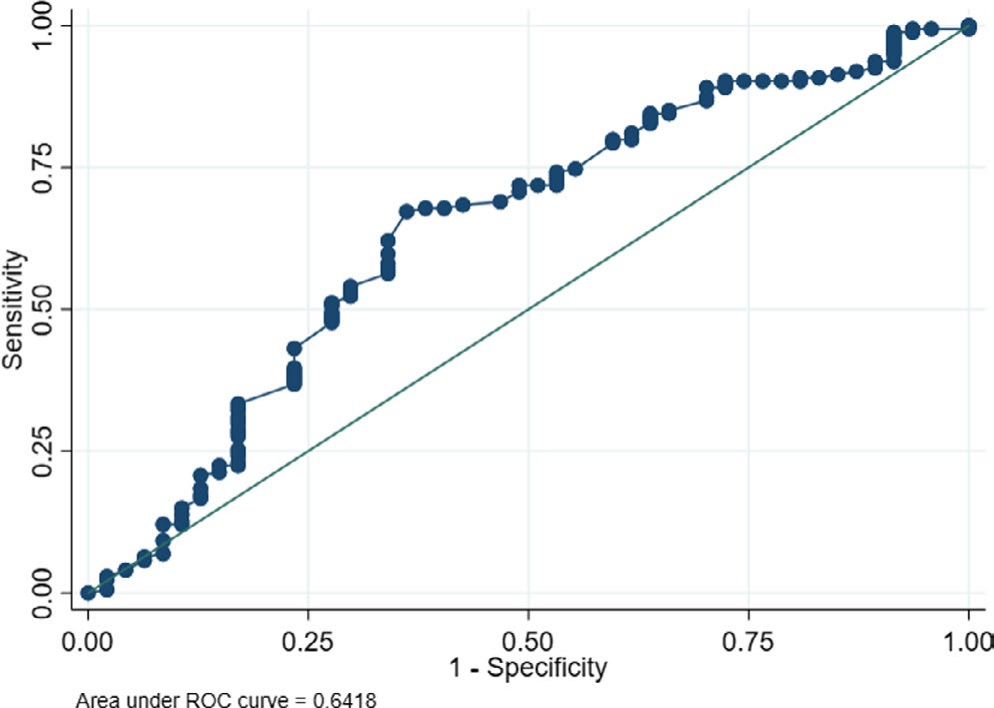
Receiver operating characteristic (ROC) curve of the performance of the predilection of *Pseudomonas aeruginosa* in diabetic foot infection

The clinicians predicted the following: the clinical involvement of *Pseudomonas* correctly in seven cases (true‐positive); its absence correctly in 167 episodes (true‐negative); its presence incorrectly in 32 cases (false‐positive); or incorrectly missed the *Pseudomonas* in 15 cases (false‐negative). Thus, the calculated performance characteristics for identifying *Pseudomonas* in the entire study population were as follows: sensitivity, 0.32; specificity, 0.84; positive predictive value, 0.18; and negative predictive value, 0.92. If we consider true‐positive and true‐negative predictions as correct, then the combined proportion of correct prediction was 79% (174/221 episodes). The comparison between the visual and olfactory predictions in terms of the presence of *P*.* aeruginosa* in DFIs revealed similar performances (8/30 vs 8/20; *p* = .48). The reported correct performance of the olfactory prediction on the presence of *P*.* aeruginosa* by female HCWs did not differ from their male homologues (2/6 vs 6/10; *p* = .53). Figure 2 depicts the corresponding receiver operating characteristic (ROC) curve, which demonstrates a moderately useful area under the ROC curve value of 0.64. The stratified predictions for *Pseudomonas* are resumed in Table 1.

**TABLE 1:** Performance characteristics of predictions of the involvement of *Pseudomonas aeruginosa* in a diabetic foot infection (*with stratifications*)

*n* = 221	Sensitivity	Specificity	Positive predictive value	Negative predictive value
Overall prediction in the entire study group	0.32	0.84	0.18	0.92
Before restitution	0.38	0.82	0.28	0.88
After the 1st restitution of results	0.22	0.85	0.12	0.94
Prediction in osteomyelitis cases only	0.20	0.81	0.09	0.92
Prediction by female healthcare workers only	0.40	0.82	0.18	0.94
Prediction by surgeons only	0.36	0.84	0.25	0.90
Cases with Gram‐negative rods seen on Gram‐stained smear	0.60	0.71	0.50	0.79

### 3.4 Improvement of prediction over time?

**FIGURE 3:**
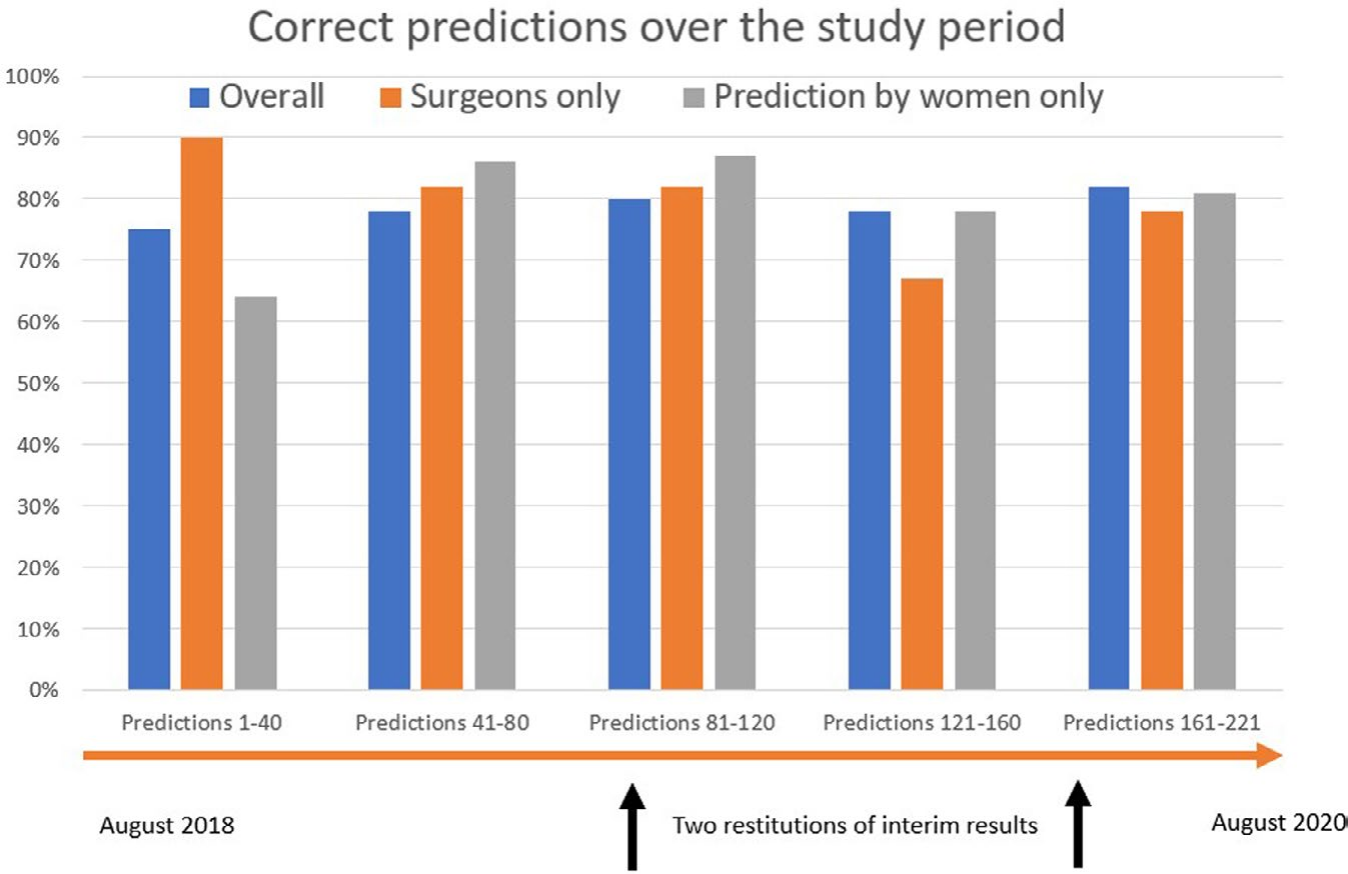
The proportions of the correct prediction of *Pseudomonas aeruginosa* in diabetic foot infections (vertical axis) over the study period. Horizontal axis; stratified in blocks of 40 consecutive episodes. The arrows indicate the timing of the feedbacks of the interim results

We assessed the overall prediction performance over the 2‐year study period in three ways: by stratifying between baseline values and those after the 1st and then the 2nd presentations of the interim results to the HCWs, and by dividing the entire study into consecutive blocks of forty or fifty predictions. Neither the presentations nor the time spent seeing patients for the study was associated with improvement in the accuracy of prediction of the presence of *Pseudomonas* in the DFI (Table 1; Figure 3). The P‐for‐trend results were negative, with a *p* = .44 for the blocks of 40 predictions and with a *p* = .46 for 50 consecutive episodes, respectively.

### 3.5 Variables associated with a correct prediction

Besides the clinical prediction in different stratifications, we wondered whether any single variables would be significantly associated with the correct (true‐positive and true‐negative) prediction. Our findings on potential associations of a variety of factors with isolation of *P*.* aeruginosa* are shown in Table 2. In Table 3, we adjusted for the case mix and provide the results of univariate and multivariate logistic regression analyses with the outcome “correct prediction.” We found that none of these variables was more associated with isolation of *Pseudomonas* than the others. Of note, because the variables “nurse” and “female HCW” revealed a significant interaction, we chose the variable “female” for the final model. However, in another multivariate run of the same model, the variable “nurse” (without the variable “female”) again failed to show a significant correlation with the outcome “correct prediction” (*data not shown*). The nonsignificant goodness‐of‐fit‐test validated our final multivariate model (*p* = .41).

**TABLE 2:** Associations with a correct prediction (true‐positive or true‐negative results) for *Pseudomonas aeruginosa* involvement in a diabetic foot infection

*n* = 221	Wrong prediction	*p* value	Correct prediction
	*n* = 47		*n* = 174
Prior antibiotic use	17 (36%)	.83	60 (34%)
Female patient	5 (11%)	.12	36 (21%)
Prediction by female healthcare worker	14 (30%)	.95	51 (29%)
Prediction by surgeon	19 (40%)	.92	69 (40%)
Osteomyelitis cases only	29 (62%)	.28	92 (53%)
After 1st restitution of interim results	16 (34%)	.40	67 (39%)
After 2nd restitution of interim results	10 (21%)	.62	43 (25%)
Gram‐negative bacteria on Gram stain	10 (21%)	.11	21 (12%)
Presence of other Gram‐negative bacteria in culture	15 (32%)	.11	36 (21%)

**TABLE 3:** Results of logistic regression analyses of the correct prediction of *Pseudomonas aeruginosa* involvement in a diabetic foot infection by associated factors

Factor (*n* = 221)	Univariate (OR, 95% CI)	Multivariate (OR, 95% CI)
Prior antibiotic use	0.9, 0.5–1.8	1.0, 0.5–1.9
Female patient	2.2, 0.8–5.9	n.d.
Prediction by female healthcare workers	1.0, 0.5–2.0	1.5, 0.6–3.9
Prediction by surgeon	1.0, 0.5–1.9	0.8, 0.4–1.6
Durations of specific professional experience (in years)	1.0, 0.9–1.1	1.0. 0.9–1.1
Presence of osteomyelitis	0.7, 0.4–1.3	0.6, 0.3–1.3
After 1st restitution of results	1.4, 0.7–2.9	1.5, 0.7–3.3
After 2nd restitution of results	1.4, 0.6–3.3	1.3, 0.5–3.4
Gram‐negative bacteria on Gram stain	0.5, 0.2–1.2	0.7, 0.3–1.7
Presence of other Gram‐negative bacteria in culture	0.5, 0.2–1.2	0.6, 0.3–1.4

Abbreviations: CI, confidence intervals; n.d., not done (due to lack of clinical relevance and due to reduced sample size); OR, odds ratio.

### 3.6 Value of the Gram‐stained smear

*Pseudomonas aeruginosa* is a Gram‐negative rod. During the study, the involved HCWs were unaware of the results of the Gram‐stained smear of the specimens submitted for culture. We were interested in the hypothetical knowing whether the results of the Gram stain could theoretically improve the HCWs' clinical prediction. The Gram‐stained smear showed bacteria in 73 cases (73/221; 33%): they were Gram‐positive cocci in 42 cases (19%), Gram‐negative rods in eight cases (4%) and a mix of Gram‐positive and Gram‐negative bacteria in 23 cases (10%). Hence, in only a total of 31 DFIs (31/211; 15%) cases were Gram‐negative bacteria seen, and in 21 of these episodes (21/31; 68%) was an organism other than *Pseudomonas* seen. However, the sensitivity of the presence of Gram‐negative rods on Gram stain (50%–60%) for the prediction of pseudomonal DFI was better than for any other single clinical factor (Table 1).

## 4 DISCUSSION

In our tertiary centre highly specialized in the management of DFIs, the visual and olfactory performance of experienced HCWs in predicting *Pseudomonas* involvement in DFI was moderate, with a much better specificity (approximately 80%–85%) than sensitivity (10%–20%). Furthermore, the likelihood of correctly predicting the presence of *Pseudomonas* did not improve during the 2 years we ran the study or after the HCWs making the predictions were given presentations on the preliminary results of the study. The one factor that moderately suggested it might be useful in predicting *Pseudomonas* infection was the presence of Gram‐negative rods on a Gram‐stained smear of the culture specimen. However, in our clinical study the HCWs were unaware of that staining, making this performance basing on the Gram staining only theoretical in the absence of real‐life conditions. Of note, the real overall performance was similar in all strata of HCWs studied, including specifically for surgeons, women and DFO cases. As this study included 221 predictions over 2 years, we think it is unlikely it was underpowered to detect the ability of clinical findings to predict the clinical involvement of *Pseudomonas* spp.

Considering that this is an important clinical question, it is surprising that we could find no publication that previously addressed this issue in the medical literature. There are certainly many published microbiological surveys of DFIs in various geographical settings, but none investigated the performance of the clinical factors widely used by clinicians when tailoring their initial empirical antibiotic choice. The importance of this question is clear when studies have found that *P*.* aeruginosa* is a pathogen in DFI in up to 40%–50% DFIs.^2^ While many clinicians believe that the presence of a green colour or grape juice smell portends *Pseudomonas*, the only well‐established clinical sign of *P*.* aeruginosa* infection is *ecthyma gangrenosum*.^20^ This rare and fulminant infection associated with pseudomonal sepsis is characterized by round erythematous macules and patches that develop into central pustules with surrounding erythema, then haemorrhagic vesicles and eventually a gangrenous ulcer with a black eschar.^9^ It is very rare in the DFI and usually occurs in the extremities of immune‐suppressed patients,^20^ including in children.^9^

*Pseudomonas aeruginosa* bacteria are known to produce pigments such as pyoverdine (a yellow‐green pigment; see Figure 1), pyocyanin^21^ and pyochelin (a blue‐green pigment). These can combine to produce a green colour in wounds,^8^ as well as the characteristic smell of 2‐amino‐acetophenone when present in high amounts.^22^ But, it is unclear whether these factors are clinically useful in predicting the presence of *Pseudomonas* in infected wounds. Indeed, many soft‐tissue infections, and practically all osteoarticular infections caused by *Pseudomonas*,^23^ lack the green colour. Several companies have developed expensive tools designed to help visualize the greenish colours purported to suggest the presence of *Pseudomonas* on the wound surface, or to detect the supposedly characteristic smell of *P*.* aeruginosa* with whole‐cell biosensors.^22^ Using these tools is, however, time‐consuming, cumbersome and expensive, and they have not yet been proven to be useful.^22^

We conducted this study in a large referral centre with special experience in dealing with DFIs, and enrolled a large number of patients. Furthermore, we examined many potential confounding factors that might influence the usefulness of the clinical factors we assessed. Nevertheless, we think there are eight issues that may have posed limitations for our study. *First*, predicting the causative pathogen in an infection involves a mix of various concomitant objective and subjective interpretations. While one clinician might prefer the visual aspects, another might rely on the odour or on the colour of the removed dressings. Moreover, HCWs often also rely on the patient's history (eg assuming a higher risk for *Pseudomonas* in the presence of ongoing antibiotic therapy, wound maceration or foot ischaemia). We did not solicit the exact reasons HCWs used in the prediction for every individual episode, although in our discussion with them the visual aspects were predominant.

*Secondly*, we do not consider any potential intra‐observer consistency. It would have been interesting if we had been able to determine whether the same HCW would make exactly the same prediction for a wound (eg by assessing a photograph of the wound several weeks later) in the same way. This, however, was not practicable in our study of patients undergoing routine clinical care.

*Thirdly*, we are fortunate to be in a resource‐rich care setting with a relatively low prevalence of pseudomonal DFIs (about 10%). It is possible that other teams, in resource scare settings with a higher prevalence of *Pseudomona*s infections, would have achieved a better taste of correct predictions. With the growing importance of antibiotic stewardship in managing DFI, we think determining whether the cheap and relatively easy clinical prediction of *Pseudomonas* in DFI is accurate and worth to be further investigated.^1^

*Fourthly*, we only formally presented the results of the HCW's performance to them at the two sessions conducted during the study, but we continuously performed a feedback of the results between these two time‐points. Furthermore, interested HCWs could assess the final microbiological results by themselves throughout the entire study period. Certainly, a more wholehearted educational programme, which usually comprises factors such as a multimodal approach with professional behavioural science and e‐learning, examinations, positive role models, and written documents, may have produced better predictions. However, our HCWs were not inexperienced and should not have needed substantial teaching in this assessment. For such groups, an iterative presentation seems an appropriate way to improve the individual performance.^24^ We think the more likely reason for our moderate performance is the genuine difficulty to clinically detect *Pseudomonas* spp. in the infected diabetic foot.

*Fifthly*, the study stretched over 2 years. Although we asked every HCW individually, we cannot exclude the likelihood that individuals were influenced by their peers, with whom they closely work. This may lead to a kind of “group think,” leading to the clinical prediction to undergo a “regression to the mean.” This could partially explain the similar performance among the HCWs, as shown in Figure 3.

*Sixthly*, we did not ask the patients to predict themselves. They could have served as the ultimate control group! Likewise, we did not determine whether the prediction was improved by using technical gadgets for the visual or olfactory identification of *Pseudomonas*. This would likely have strongly influenced the HCWs clinical judgement and introduced a major bias. We were interested in estimating the value of just the clinical findings, without or supplementary technical aid. The only exception was the theoretical value of the Gram‐stained smear, did appear to improve the sensitivity of prediction when Gram‐negative bacteria were identified.

*Seventhly*, our study question assumes the necessity of treating all *Pseudomonas* spp. in DFI with the correct empirical agent from the start, as one pathogen, for example the *P*.* aeruginosa*, can be a colonizer in the concomitant involvement of other true infecting pathogens. Even if our study intended to separate colonization from active clinical infection as much as possible, we cannot express on the pathogenic activity or the proportion of pseudomonal damage in polymicrobial DFIs. We additionally believe that not all pathogens need specific antimicrobial coverage in DFIs. For instance, DFIs may resolve when patients are treated with antibiotics that do not cover selected bacteria (including *Pseudomonas* ^20^ and enterococci^25^, ^26^). Certainly, experts^5^ acknowledge that every proven deep *Pseudomonas* infection of bone, and serious monomicrobial infections of the soft tissue, requires targeted antibiotic treatment,^23^, ^27^ whereas many chronic, ischaemic and polymicrobial soft‐tissue DFIs do not. Frequently, superficial *Pseudomonas* can represent colonization of maceration tissue and it can be removed by debridement alone, especially in mild DFIs.^28^

*Lastly*, our study does not address the clinical consequences of the initial microbiological diagnostic prediction. The associated key questions are basing on the harms provoked by a false‐positive or false‐negative guess of pseudomonal involvement. The negative consequences of a false‐positive prediction are clear. It contributes to an unnecessary broad‐spectrum empirical antibiotic coverage, exposing the healthcare system and the DFI patients to all deleterious aspects that antibiotic stewardship wants to avoid.^1^ A false‐negative prediction leads to a delay of 2–4 days regarding the correct antibiotic treatment or to a partial antibiotic coverage from the start. This delay can be deleterious for a minority of DFI patients, but probably only in the DFI subpopulation with severe, acute soft‐tissue infections (bacteraemia, fever, shivering or sepsis). In these patients, an empirical broad‐spectrum coverage against all nonfermenting rods is indicated, and not only because of *P*.* aeruginosa*.^4^ In contrast, in mild to moderate soft‐tissue DFIs, or in all chronic DFOs,^29^ a delay of 2–4 days before the complete targeting of all pathogens usually does not alter the overall outcome in the multifaceted setting of chronic, polymicrobial DFIs, especially not when there is a large surgical debridement. We did not yet publish our specific analogy data for our soft‐tissue DFI cases. But, as a general rule for all orthopaedic infections, a wrong or uncomplete empirical antibiotic coverage during the some few initial days, after surgical debridement and in the absence of a sepsis or bacteraemia, does not alter the remission rate after a weeks‐long targeted antibiotic therapy, which has been already published regarding various implant‐related orthopaedic infections.^30^

## 5 CONCLUSION

We attempted to answer a very important question, specifically how good are clinicians in predicting the involvement of *Pseudomonas* spp. in DFI. We used a prospective methodology that spanned a 2‐year period. Regrettably, as anticipated, clinicians are only somewhat capable at predicting the presence of *P*.* aeruginosa*. The combined (olfactory and visual) ability to predict the presence of *Pseudomonas* spp. in DFI among our experienced HCW was only moderate, with a much better specificity (80%–85%) than sensitivity. Presenting the interim results of our study to the HCWs did not improve their prediction scores. We believe the performance characteristics of these clinical signs alone are too low to use them to tailor an initial, empirical antibiotic regimen for DFIs, which should base on infection severity rather than on the pseudomonal guess. However, when all clinical findings are negative this could be used in a population with low pre‐test probability to largely rule out a *Pseudomonas* infection. The advantages of tailoring the initial empirical antibiotic coverage for pseudomonal DFI based on the results of the cheap and rapid clinical assessment remain tempting. In the light of the great need for antibiotic stewardship facing to help address the ever‐increasing problem of antibiotic resistance,^1^ we would like to see our study repeated in a high‐prevalence setting. As the positive and the negative predictive values depend on the prevalence, the benefits of the clinical prediction (perhaps with the additional help of a rapidly available Gram‐stained smear) could prove beneficial in high‐risk settings.^2^

## CONFLICT OF INTEREST

All authors declare that they have no conflict of interest with this work or any financial relationships relevant to this study.

## AUTHOR CONTRIBUTION

IU conceptualized the idea, drafted the manuscript, sponsored the study, conducted the experiments, analysed the data and wrote the manuscript. DH conducted the study, participated in patient inclusion and supervised the study. MS conducted the study, participated in patient inclusion, supervised the study and wrote the manuscript. FWA participated in patient inclusion and conducted the study. TT participated in patient inclusion, conducted the study and wrote the manuscript. JB participated in patient inclusion and supervised the study. TB conceptualized the idea, and organized and supervised the study. BAL designed the concept, wrote the manuscript and performed corrections. MB designed the concept, performed corrections, supervised the study and participated in patient inclusion.

## Supporting information

Appendix S1

Appendix S2

## Data Availability

We may share anonymized key data upon scientific request to the corresponding author.
