# Supplementary material for: How good are clinicians in predicting the presence of Pseudomonas spp. in diabetic foot infections? A prospective clinical evaluation
Source: Endocrinol Diabetes Metab. 2021 Feb 9;4(2):e00225. doi: 10.1002/edm2.225 (PMC8029573; doi:10.1002/edm2.225)
Supplement: Supplementary file 2 — Appendix S2 [file EDM2-4-e00225-s001.pdf]

Introduction

Diabetic Foot Infections (DFI): pivotal event leading to lower extremity amputation

***Pseudomonas* spp:**

- **Virulent pathogen, strong biofilm**
- **Frequent in nosocomial infections**
- **Resistant to standard antbiotics used as first – line therapy for DFI**
- **May become rapidly resistant during therapy**
- **"Recognizable": green colour, grape juice – like smell, skin maceration ?**

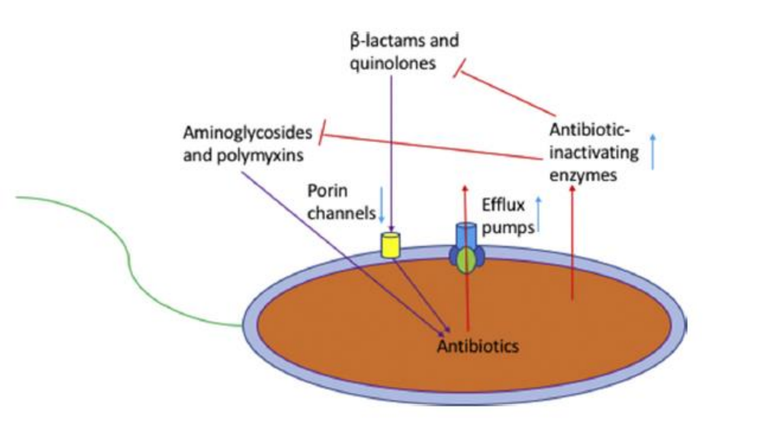

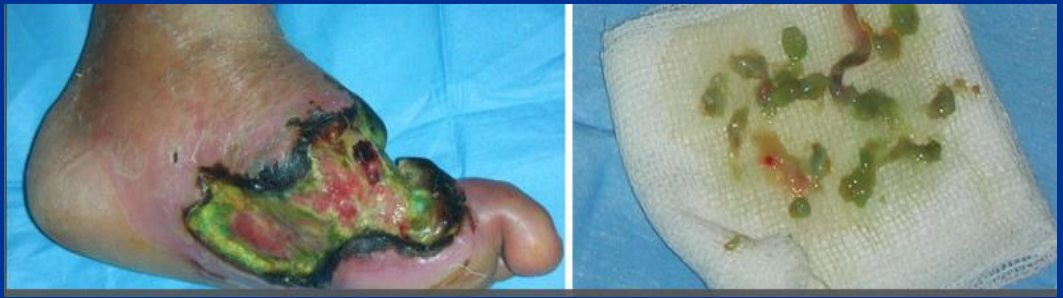

Prompers et al. Diabetologia 2008

Uckay et al. Diabetes, Obesity and Metabolism 2014

Pang et al. Biotechnology Advances 2019

First author [Reference]	Country	Year	Types of wounds	No. of patients	Percentage of isolates from wound culture					
					Staphylococci	Streptococci	Gram-positive	Gram-negative	*Ps. aeruginosa*	Anaerobes
Carvalho [109]	Brazil	2003	Infections	141	20	4	29	59	7	12
Candel [110]	Spain	2003	Infections	27	49	15	78	22	1	2
Anandi [111]	India	2004	Infections	107	14	—	—	—	—	4
Unachukwu [112]	Nigeria	2005	Gangrene	60	56	—	—	—	—	—
Senneville [113]	France	2005	Bone	76	52	12	—	18	2	5
Abdulrazak [114]	Kuwait	2005	Infections	86	38	17	74	26	18	11
Shankar [115]	India	2005	Infections	77	—	3	42	58	30	6
Yoga [116]	Malaysia	2006	Infections	44	20	—	—	—	14	—
Gadepalli [72]	India	2006	Ulcers	80	20	0	33	51	10	15
Sharma [117]	Nepal	2006	Ulcers	—	38	—	—	—	18	—
Őrmen [118]	Turkey	2007	Bone	50	—	—	40	60	—	—
Raja [119]	Malaysia	2007	Infections	194	44	25	45	52	25	—
Çetin [120]	Turkey	2007	Infections	65	18	6	59	41	8	3
Dowd [121]	USA	2008	Ulcers	40	8	37	—	—	15	18
Umadevi [122]	India	2008	Infections	105	17	0	29	71	17	0
Khoharo [123]	Pakistan	2009	Infections	60	20	3	27	73	48	2
Ramakant [35]	India	2010	Ulcers	447	19	3	31	57	17	1
Zubair [124]	India	2010	Infections	60	31	0	38	62	11	0
Őzer [43]	Turkey	2010	Infections	78	17	7	38	56	19	—
Mendes [125]	Portugal	2011	Infections	49	84	4	85	19	1	14
Hayat [126]	Pakistan	2011	Infections	85	18	5	27	68	27	2
Pappu [127]	India	2011	Infections	104	21	4	—	>67	23	0
Malone [128]	Saudi-Arabia	2011	Toe bone	34	33	9	57	29	9	0
Aziz [129]	Singapore	2011	Infections	100	40	21	—	—	26	30
Dezfulian [138]	Iran	2011	Infections	69	43	5	55	45	6	5
Aamir [36]	Pakistan	2011	Wounds	114	55	5	—	—	4	—
Tiwari [130]	India	2012	Infections	62	—	—	32	68	—	—
Swarna [131]	India	2012	Infections	62	30	0	44	56	20	0
[illegible]	[illegible]	[illegible]	[illegible]	[illegible]	[illegible]		[illegible]	[illegible]	[illegible]	

of

Study Purpose and Design

To investigate the capacity of experienced clinical workers to predict *Pseudomonas* spp. involvement in infected foot ulcers based on typical clinical findings (green color, specific smell, skin maceration ?)

Prospective observational pilot study

Interim Results

Growth of *Pseudomonas* in 13 cases (15.2%)

- **7 bone samples (8.2%)**
- **4 mono – infections**
- **9 co - infections**

Clinicians predicted *Pseudomonas:*

- **Correctly in 5 cases**
- **Wrongly in 13 cases**
- **Missed it in 8 cases**

	Overall:	Surgeons:
Sensitivity	0.38	0.50
Specificity	0.83	0.94
Positive predictive value	0.29	0.75
Negative predictive value	0.88	0.84

Conclusion

The predictive capacity for *Pseudomonas* spp. according to visual and olfactory characteristics in DFI was moderate even among experienced clinicians

Better specificity than sensitivity

Effect of awareness / training (restitution) over time, the role of the Gram-staining and performance of wound nurses need to be further elucidated

Microbiology is still necessary
